# Neutrophil Extracellular Traps Identification in Tegumentary Lesions of Patients with Paracoccidioidomycosis and Different Patterns of NETs Generation In Vitro

**DOI:** 10.1371/journal.pntd.0004037

**Published:** 2015-09-01

**Authors:** Amanda Manoel Della Coletta, Tatiana Fernanda Bachiega, Juliana Carvalho de Quaglia e Silva, Ângela Maria Victoriano de Campos Soares, Julio De Faveri, Silvio Alencar Marques, Mariângela Esther Alencar Marques, Valdecir Farias Ximenes, Luciane Alarcão Dias-Melicio

**Affiliations:** 1 Department of Pathology, UNESP – São Paulo State University, Botucatu Medical School, Botucatu, São Paulo, Brazil; 2 Department of Microbiology and Immunology, UNESP—São Paulo State University, Biosciences Institute, Botucatu, São Paulo, Brazil; 3 Department of Dermatology and Radiotherapy, UNESP – São Paulo State University, Botucatu Medical School, Botucatu, São Paulo, Brazil; 4 Department of Chemistry, UNESP—São Paulo State University, School of Sciences, Bauru, São Paulo, Brazil; University of California San Diego School of Medicine, UNITED STATES

## Abstract

Paracoccidioidomycosis (PCM) is a systemic mycosis, endemic in most Latin American countries, especially in Brazil. It is caused by the thermo-dimorphic fungus of the genus *Paracoccidioides* (*Paracoccidioides brasiliensis* and *Paracoccidioides lutzii*). Innate immune response plays a crucial role in host defense against fungal infections, and neutrophils (PMNs) are able to combat microorganisms with three different mechanisms: phagocytosis, secretion of granular proteins, which have antimicrobial properties, and the most recent described mechanism called NETosis. This new process is characterized by the release of net-like structures called Neutrophil Extracellular Traps (NETs), which is composed of nuclear (decondensed DNA and histones) and granular material such as elastase. Several microorganisms have the ability of inducing NETs formation, including gram-positive and gram-negative bacteria, viruses and some fungi. We proposed to identify NETs in tegumentary lesions of patients with PCM and to analyze the interaction between two strains of *P*. *brasiliensis* and human PMNs by NETs formation *in vitro*. In this context, the presence of NETs *in vivo* was evidenced in tegumentary lesions of patients with PCM by confocal spectrum analyzer. Furthermore, we showed that the high virulent *P*. *brasiliensis* strain 18 (Pb18) and the lower virulent strain Pb265 are able to induce different patterns of NETs formation *in vitro*. The quantification of extracellular DNA corroborates the idea of the ability of *P*. *brasiliensis* in inducing NETs release. In conclusion, our data show for the first time the identification of NETs in lesions of patients with PCM and demonstrate distinct patterns of NETs in cultures challenged with fungi *in vitro*. The presence of NETs components both *in vivo* and *in vitro* open new possibilities for the detailed investigation of immunity in PCM.

## Author Summary

Paracoccidioidomycosis (PCM) is an infectious disease caused by fungi of genus *Paracoccidioides* (*P*. *brasiliensis* and *P*. *lutzii*). PCM is endemic in Latin America, with a greater incidence in Brazil, Colombia, and Argentina. Over the last years, studies are focusing on neutrophils’ (PMNs) actions against *P*. *brasiliensis*, due to the capacity of these cells to develop different defense strategies against pathogens. and especially due to constant presence of inflammatory infiltrates full of PMNs in the granuloma of the disease. As PMN release of both granular and nuclear material, identified as Neutrophil Extracellular Traps (NETs), is a spectacular action mechanism against microbes, we seek to identify whether this process would be an important mechanism triggered against *P*. *brasiliensis*. Thus, we showed for the first time the identification of NETs in tegumentary lesions of patients with PCM by viewing the individual components of NETs. Beyond that, we demonstrated the entrapment of *P*. *brasiliensis in vitro* by these structures released from human PMNs of patients with PCM and healthy donors, with different patterns, in a dependence of the evaluated strain. Our data provides important new information regarding the role of PMNs against *P*. *brasiliensis*, opening new avenues for the research on immunity of PCM.

## Introduction

Paracoccidioidomycosis (PCM) is a systemic mycosis considered an important cause of mortality and morbidity in most Latin American countries, especially in Brazil. Sporadic cases have been reported in European countries, United States of America (USA) and Japan, in individuals coming from endemic areas [1–5]. It is caused by the fungi of the genus *Paracoccidioides* (*Paracoccidioides brasiliensis* and *Paracoccidioides lutzii*) [6,7], which share the same thermo-dimorphic features, developing as mycelium at room temperature and as yeast at body temperature [8,9]. *P*. *brasiliensis* infection occurs after propagules inhalation (conidia presented in water, soil and plants) [10,11], which are deposited in the lungs and transformed into yeast cells, establishing the disease. From this stage on, infection could become latent (PCM–infection), disseminate by lympho-haematogenic pathway to other organs, such as liver and spleen (PCM–disease), or heal spontaneously [11].

Innate immune response is essential during early stages of fungal infections [12]. Phagocytic cells, such as neutrophils (PMNs) and macrophages, play crucial role in host defense, modulating the inflammatory response and fungicidal activity against *P*. *brasiliensis* [12–17]. In this context, studies have focused on the role of PMNs during PCM, since a massive infiltration of these cells is found in granulomas of the disease, after chemoattraction modulated by keratinocyte chemoattractant (KC) and macrophage inflammatory protein 1 alpha (MIP-1α) [18].

PMNs are short-lived cells that must be promptly recruited to the site of infection [19]. They can capture and kill microbes by oxygen dependent or independent mechanisms, by the action of NADPH enzyme or release of their granular components [19]. Reactive oxygen species (ROS), produced by the action of NADPH enzyme are essential for the killing of fungi [14,15,20–23]. Previous studies demonstrated that non-activated PMNs do not have fungicidal activity, just showing fungistatic activity against *P*. *brasiliensis* [24], with an increase in these functions after activation with cytokines such as interferon-gamma (IFN-γ), tumor necrosis factor-alpha (TNF-α), granulocyte monocyte colony-stimulating factor (GM-CSF) and interleukin-15 (IL -15) [24–27]. The studies also showed that the effector mechanisms of activated PMNs against fungi involve superoxide anions and H_2_O_2_ participation.

A novel PMN mechanism of action has been described as NETosis, which is an extracellular mechanism to kill microbes characterized by the PMN release of both granular and nuclear material and identified as Neutrophil Extracellular Traps (NETs) [28]. These structures are composed by a decondensed DNA backbone associated with histones and others antimicrobial proteins such as elastase, permeability increasing protein (BPI) and myeloperoxidase [28,29]. NETs can be triggered by gram-positive and gram-negative bacteria, fungi, protozoa and viruses, some molecules like interleukin-8 (IL-8), Phorbol Myristate Acetate (PMA), lipopolysaccharide (LPS) and others cells as activated platelets [28,30–34], showing until now, that several microorganisms are able to induce NETs formation. In some of them, NETs have antimicrobial activities, in others meanwhile, these structures have only temporary entrapment action, avoiding their dissemination [28,31,33–37].

Therefore, the aims of this study were to identify the presence of NETs *in vivo*, analyzing tegumentary lesions of patients with PCM, and *in vitro*, challenging human PMNs with *P*. *brasiliensis* yeast cells.

## Materials and Methods

### Casuistics

A prospective study was conducted to analyze skin tegumentary lesions of seven male patients between 51 and 75 years old, attended at clinical dermatology of the Botucatu Medical School, São Paulo State University. All patients had the chronic form of PCM with lesions localized at head, nose, hand, knee, foot and back. The diagnosis was confirmed by histopathological analysis performed by the Pathology Service/FMB. Patients were selected before treatment, excluding the immunocompromised ones and those with secondary infections.

PMNs from peripheral blood of PCM patients with the chronic form of the disease and healthy volunteer donors between 20 and 30 years from FMB were also evaluated in this study.

### Ethics statement

This investigation was conducted according to the principles expressed in the Declaration of Helsinki and was approved by the Research Ethics Committee of Botucatu Medical School, UNESP–São Paulo State University (CEP—261/11). Written informed consent was obtained from all participants.

### Isolation, purification and culture of human peripheral blood PMNs

Peripheral blood from patients and healthy donors was collected by venous puncture and PMNs were separated by a density gradient centrifugation (Histopaque 1119 and 1083g/mL—Sigma–Aldrich, St. Louis, USA) at 460 g for 30 minutes followed by erythrocytes lysis with a hypotonic solution (NaCl 0,2%). Cellular viability was assessed by trypan blue dye exclusion test, and purified PMNs (≥95% of the cells) were then resuspended in complete medium (RPMI medium 1640 supplemented with 10% inactivated fetal calf serum, both from Sigma–Aldrich) and placed on ice until use. Cell culture was adjusted for 2x10^6^ cells/mL before all procedures.

### Fungi

Two different strains of *P*. *brasiliensis* were used throughout this study: *P*. *brasiliensis* strain 18 (Pb18, high virulence) and strain 265 (Pb265, low virulence). The strains were submitted to weekly sub-cultivation on 2% glucose, 1% peptone, 0.5% yeast extract and 2% agar medium (GPY medium) (all reagents from DIFCO, Franklin Lakes, NJ, USA), and used on the sixth day of culture. For preparation of *P*. *brasiliensis* suspension, yeast cells were removed from the cultivation medium, transferred to a sterile test tube containing glass beads and homogenized in a Vortex homogenizer (two cicles of ten seconds). Yeast viability was determined by phase contrast microscopy and bright yeast cells were counted as viable while dark ones were considered as non-viable. Fungal suspensions containing more than 95% viable cells were used in the experiments. The yeast suspension was adjusted for 4x10^4^ cells/mL before use.

### Histopathological analysis

Tissue sections from biopsies of tegumentary lesions from seven PCM patients were fixed with buffered formalin, dehydrated in 70% alcohol and embedded in parafin. Samples (7μm thick) were deparaffinized and stained with hematoxylin and eosin (H&E), in attempt to identify the extracellular DNA, representative of NETs [38]. Sections of the same biopsies were stained with Gomori-Grocott to visualize yeast cells. Gomori-Grocott stain consists in oxidizing the sample with chromic acid 5%, bleaching with sodium bisulphite 1%, treating with methenamine silver solution until impregnation, toning with gold chloride 0.1%, fixing with sodium thiosulphate 3% and counterstaining with light green. Slides were scanned with the Pannoramic Digital Slide Scanners—Pannoramic MIDI (3DHISTECH Kft, Budapest, Hungary) and analyzed using software Pannoramic Viewer 1.15.4 RTM (3DHISTECH Kft), from NTDP (Digital Technologies in Pathology Facility, Dept. of Pathology–FMB).

### NETs analysis by confocal laser scanning microscopy

The same biopsies were analyzed by confocal laser scanning microscopy. Tissue sections (7μm thick) were deparaffinized in two baths of 100% xylene and three baths of ethanol decreasing concentrations (100%, 90% and 70%). Samples were washed for 5 minutes in dH_2_O and incubated with a blocking buffer PBS-BSA 4% (nonspecific binding block) for 30 minutes. Tissues were incubated with anti-elastase (Calbiochem—Merck Millipore—Merck KGaA, Darmstadt, Germany) and anti-histone H1 (Millipore—Merck Millipore—Merck KGaA, Darmstadt, Germany) antibodies, followed by anti-rabbit-FITC (Millipore) and anti-mouse-Texas red (Calbiochem) antibodies, respectively. Slides were mounted using mounting medium for fluorescence with DAPI (Vectashield-Vector Labs, Burlingame, CA, USA).

Confocal images were taken in a Leica TCS SP5 microscope from the CME (Electron Microscopy Center–Biosciences Institute—UNESP—Botucatu).

### NETs analysis by scanning electron microscopy

Isolated PMNs (2x10^6^ cells/mL) from PCM patients and healthy donors were adhered on coverslips treated with Poly-L-Lysine 0, 01% (Sigma-Aldrich) in 24-well flat-bottom plates (Nunc Life Tech., Inc., MD, USA). In some cultures, after adherence, cells were pretreated with DNAse (100U/mL–Fermentas Life Science–St. Leon-Rot, Germany) for 30 minutes and/or PMA (100ng/mL- Sigma–Aldrich) as a negative and positive control respectively. PMNs were then challenged with Pb18 and Pb265 (4x10^4^ cells/mL), using 50:1 cells/fungi ratio, and incubated for one or two hours in 5% CO_2_ at 37°C. Cocultures were fixed with 2.5% glutaraldehyde in 0.1 M cacodylate buffer, pH 7.2, postfixed with 1% osmium tetroxide, and dehydrated with an ascending ethanol series. After dehydration and critical-point drying, samples were coated with gold and analyzed in a FEI QUANTA 200 scanning electron microscope from the CME (Electron Microscopy Center–Biosciences Institute—UNESP—Botucatu).

### High-content image acquisition and analysis of NETs

PMNs isolated from healthy donors were treated as described previously. For automated imaging acquisition, isolated PMNs (2x10^6^ cells/mL) were treated with PMA–positive control (100 ng/mL–Sigma Aldrich) for 30 minutes or challenged with Pb18 or Pb265 (4x10^4^ cells/mL – 50:1 cells/fungi ratio) and incubated for two hours in 5% at 37°C. After this period, cells were stained with anti-elastase antibody (Calbiochem), followed by anti-rabbit-FITC antibody (Millipore), fixed with 4% formaldehyde solution and DNA was stained with DAPI (Vectashield–Vector Labs). Following this, cultures were transferred into 96-well black/clear bottom microplate (Corning) at 2 × 10^5^ cells/well in 100 μl microplates and were centrifuged at 2000 rpm for 5 minutes at 24°C. Images were acquired using the automated microscope ImageXpress Micro XL Widefield High-Content Screening System (Molecular Devices, Sunnyvale, CA) with a cooled 16-bit monochromes CMOS PCO camera (2160 × 2160 imaging array, 6.5 × 6.5 μm pixels) using an 20× Super Plan Fluor ELWD, NA 0.45 Nikon objective. Exposure times were 200 msec for DAPI (nuclear stain) and 500 msec for FITC (anti-elastase-FITC antibody). A total of 16 sites for each replicate (n = 5) was acquired from wells containing non-treated PMNs, 100 ng/ml PMA-activated cells, and PMNs incubated with Pb18 or Pb265 (50:1 ratio). Cellular image analysis was performed using MetaXpress software version 5.3.0.5. Average of total DNA stained by DAPI was quantified using a customized method with MetaXpress Custom Module Editor. Total area (μm^2^) of the resulting NETs in response to Pb18 and Pb265 was analyzed by thresholding NETs stained with DAPI and anti-elastase-FITC antibody and measured by Integrated Morphometry Analysis.

### Quantification of NETs release by Picogreen dsDNA kit

PMNs (2x10^6^ cells/mL) from healthy donors were incubated with or without Pb18 and Pb265 for two hours. In some assays, cultures were activated with PMA–positive control (100ng/mL—Sigma–Aldrich) or challenged with Pb18 and Pb265. After incubation, supernatants were collected and treated with restriction enzymes (EcoR1 and HindIII, 15U/uL each; Invitrogen, Eugene, Oregon, USA), according to the manufacturer's instructions. After, extracellular DNA amounts (NETs) were quantified using the Picogreen dsDNA kit (Invitrogen) used by several authors [33,34,39–41]. The λ-DNA standard provided with the kit (100μg/mL) was diluted with Tris-EDTA (TE buffer) to the concentration of 1ng/mL for the high range curve and received the same treatment with restriction enzimes. Plates were incubated at room temperature in the dark for 5 minutes prior reading on a SpectraMax M2 (Molecular Devices) using an excitation wavelength of 480 nm and emission wavelength of 520 nm.

### Statistical analysis

Measure of NETs area was analyzed by Wilcoxon’s test. Average of total DNA structures and quantification of extracellular DNA were compared by Friedman’s test followed by post-hoc Dunn’s test with the level of significance set at p<0.05. All results were analyzed using GraphPad Prism 5.01 Software (Graphpad Software Inc., CA, USA).

## Results

### NETs identification by histopathological analysis

**Fig 1:**
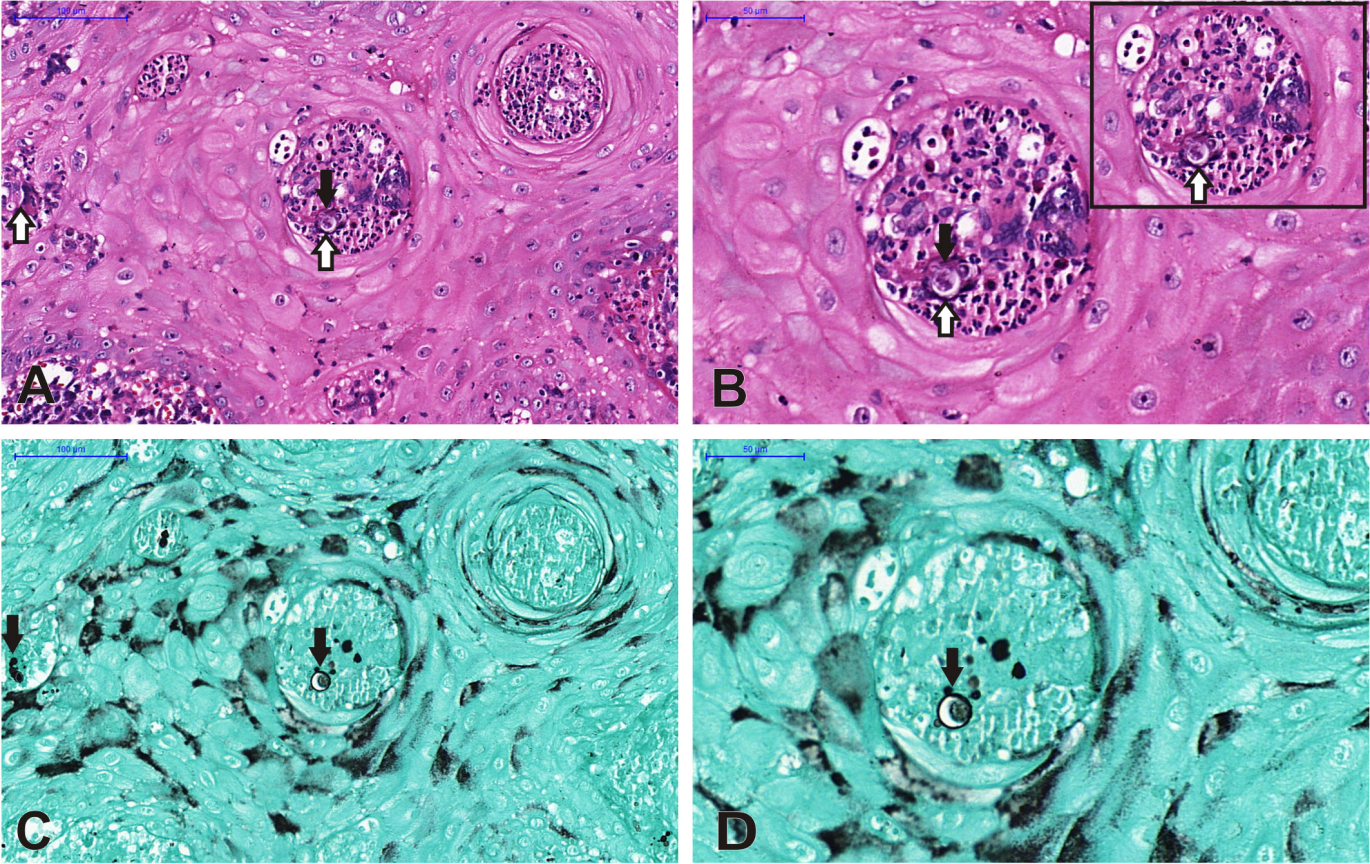
Histopathology of cutaneous lesions of patients with PCM showing Hematoxilin filamentous suggestive of NETs (white arrows) (A-20x and B-35x, insert-40x). The same sample was stained with Gomori-Grocott, confirming the presence of *P*. *brasiliensis* in the lesion (black arrows) (C-20x and D-35x). (Bar size: A and C—100μm, B and D—50μm).

In attempt to identify extracellular structures suggestive of NETs, histopathological analysis of skin tegumentary lesions of patients with PCM was performed. In Fig 1A and 1B, the image revealed basophilic material with characteristic extracellular filaments, indicating extracellular DNA (positive for hematoxylin), suggestive of the formation of NETs, which are surrounding the yeasts at the lesion. Details of NETs and yeast cell were demonstrated in the highlighted area in Fig 1B (40x). In Fig 1C and 1D, the same tissue section was stained with Gomori-Grocott, as a positive control of the presence of *P*. *brasiliensis* in the site of lesion, corroborating the idea of the fungal participation on this process.

### Individual components of NETs visualization by confocal laser scanning microscopy

**Fig 2:**
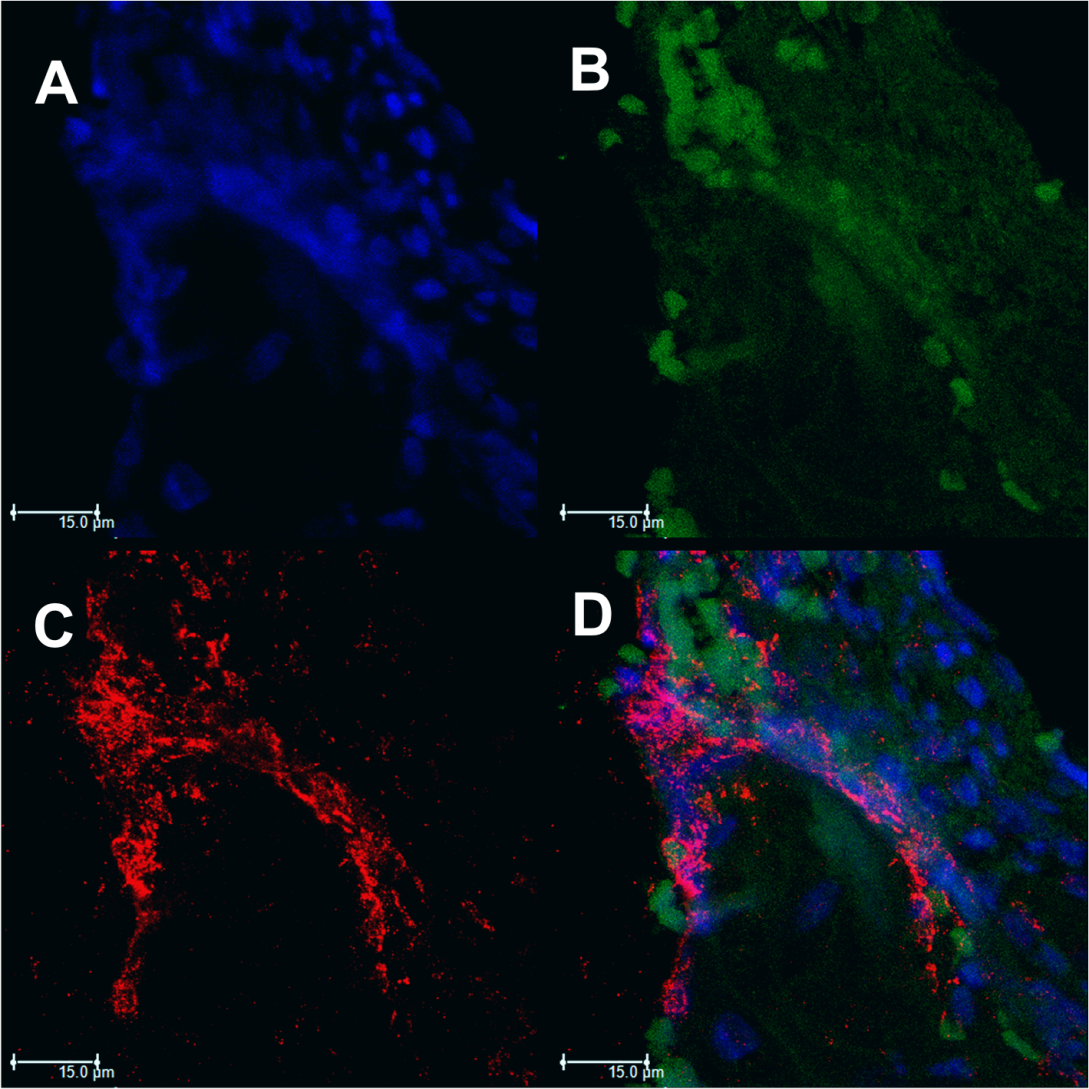
Confocal microscopy of NETs identified in skin lesions of patients with PCM. Tissue was stained with DAPI (A), labeled with anti-elastase antibody followed by FITC-conjugated secondary antibody (B) and anti-histone H1 secondary antibody followed by Texas Red (C). In the last frame, the overlapping images (D). (Bar size 15μm).

After identification of the extracellular material suggestive of NETs by histopathological analysis, we seek to identify the individual components of NETs in biopsies from tegumentary lesions of patients with PCM (Fig 2). As expected, NETs were also visualized in these samples, and their constituents were evaluated individually. Decondensed DNA, the backbone of these structures, was identified after DAPI staining (Fig 2A). Histones (Histone H1) and elastase, other major NETs components, were identified combined with DNA after immunostaining with specific primary and secondary antibodies (Fig 2B and 2C). Interestingly, Fig 2D is the overlay of these three images, confirming the co-existence of the three major components constituting the NETs.

**Fig 3:**
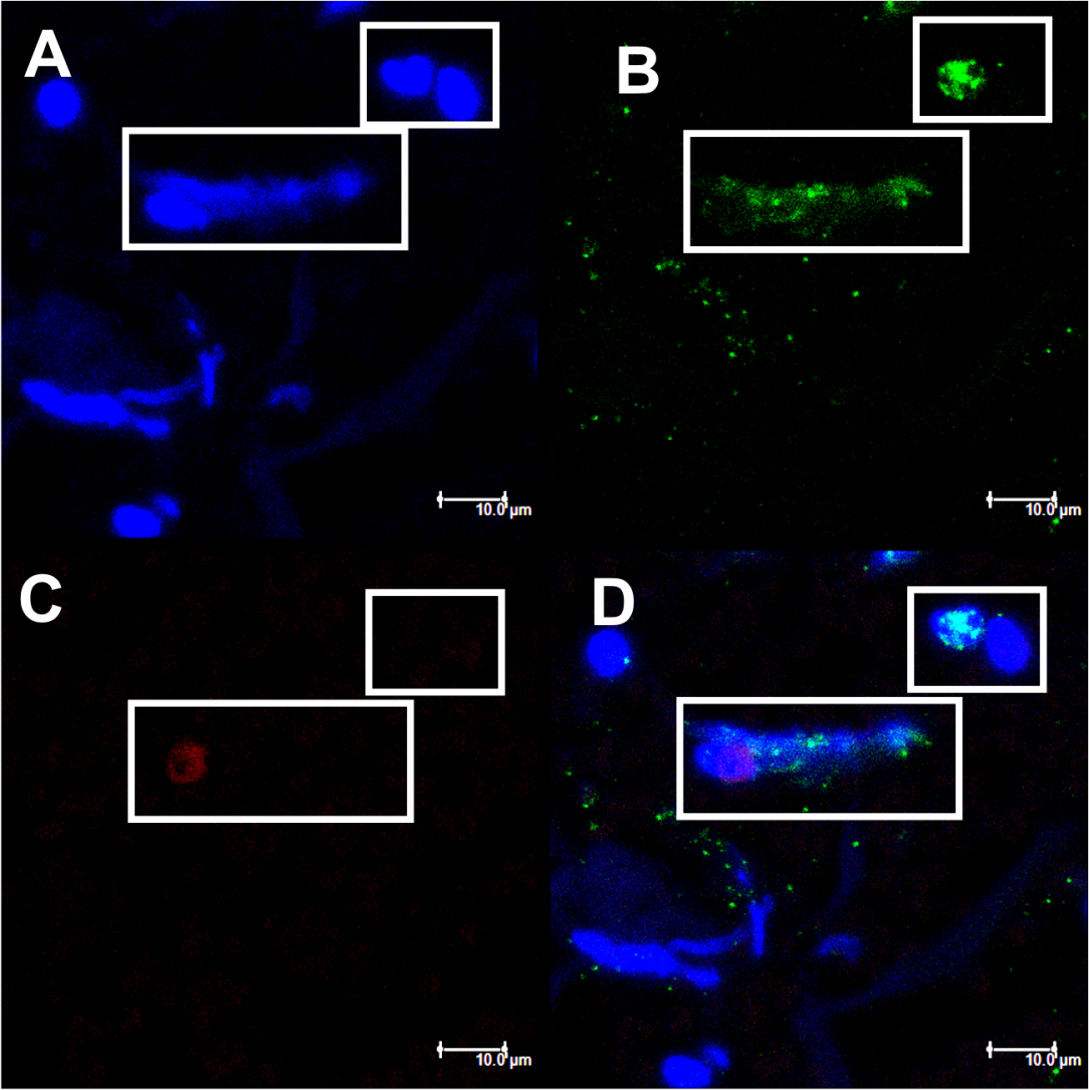
Confocal microscopy of NETs identified in skin lesions of patients with PCM. Tissue was stained with DAPI (A), labeled with anti-elastase antibody followed by FITC-conjugated secondary antibody (B) and anti-histone H1 secondary antibody followed by Texas Red (C). In the last frame, the overlapping images (D). The highlights show the process of NETs formation. (Bar size 10μm).

We also identified during the analysis of one section (Fig 3), two cells that appear to be in an early stage of NETs formation. In the box (highlighted), we identified a nucleus labeled with DAPI (Fig 3A), which had lost its normal format and had positive staining for elastase (Fig 3B), demonstrating the colocalization of elastase with nuclear DNA. Elastase is transported to the nucleus, acting on histones to initiate the chromatin decondensation, even before it is released into the cytoplasm [42]. At the center of the field, we can observe a cell that appears to be releasing their decondensed DNA content (labeled with DAPI) (Fig 3A), already bound to elastase (Fig 3B), and the histone H1 in nuclear region (Fig 3C), indicating the mobilization of these compounds in the formation of NETs. In Fig 3D, the overlap of 3 images was observed. These images are very interesting as they have been identified in lesions of patients showing an active response of human PMNs through NETs formation against *P*. *brasiliensis in vivo* for the first time, once the fungus was identified by Gomori-Grocott in the lesion.

### NETs identification by scanning electron microscopy

To visualize NETs formation *in vitro* by scanning electron microscopy, PMN cultures from patients and healthy donors were challenged with two strains of *P*. *brasiliensis*: Pb18 (high virulence strain) and Pb265 (low virulence strain).

**Fig 4:**
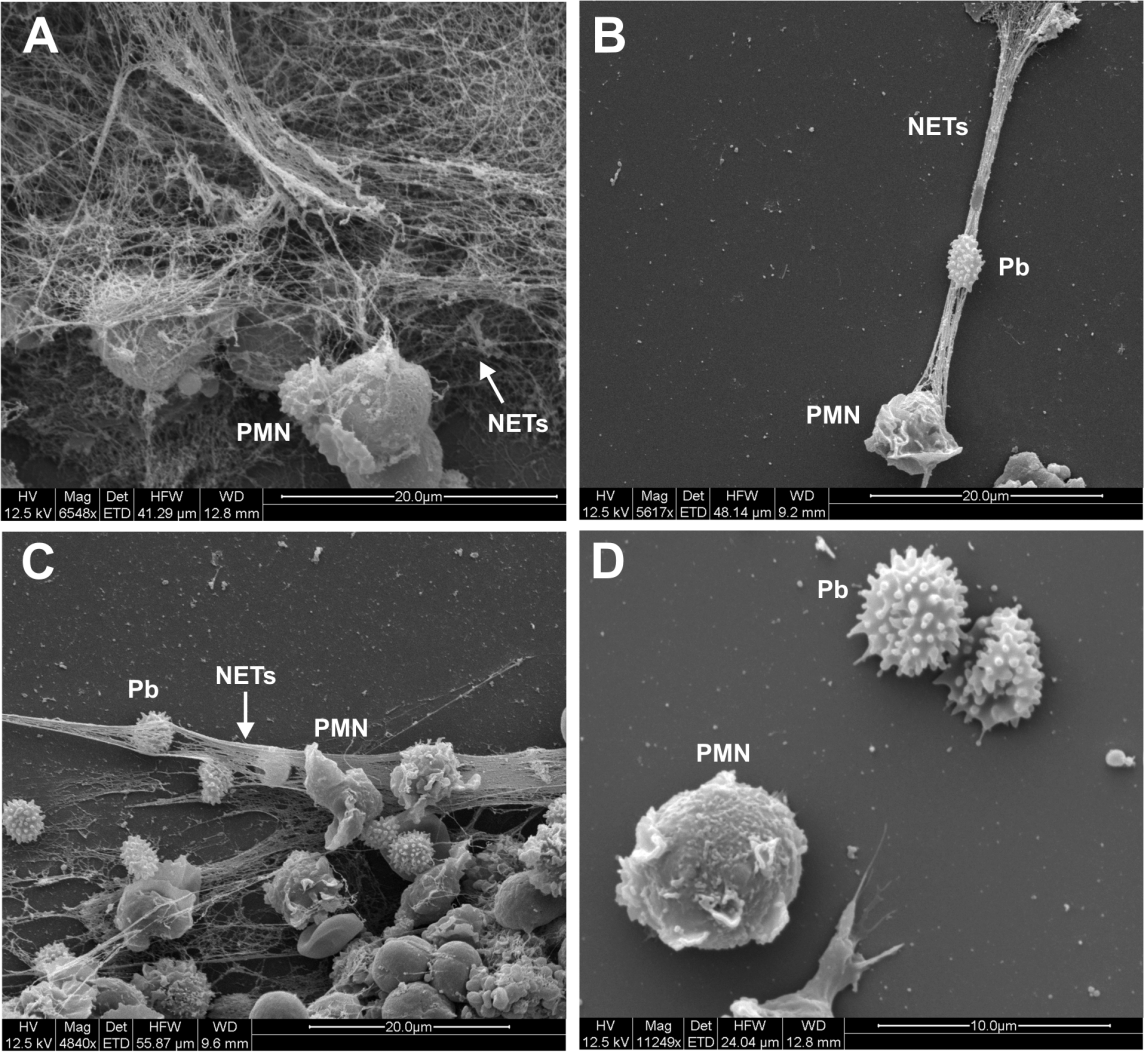
Scanning electron microscopy from PMNs challenged with *P*. *brasiliensis* (50:1 ratio) in different periods of incubation showing NETs release. (A) PMNs activated with PMA (100ng/mL) for 30 minutes. (B) PMNs challenged with Pb18 for one hour. (C) PMNs challenged with Pb18 for two hours. (D) PMNs challenged with Pb18 and treated with DNAse (100U/mL) for 30 minutes. (PMN–neutrophil; Pb–*P*. *brasiliensis*; NETs–Neutrophil Extracellular Traps).

Non-treated PMNs from healthy donors were not able to release NETs, but in the presence of PMA, these structures were formed and visible after 45 minutes of incubation. PMA was able to induce an intense release of NETs that completely covered the observed area (Fig 4A).

**Fig 5:**
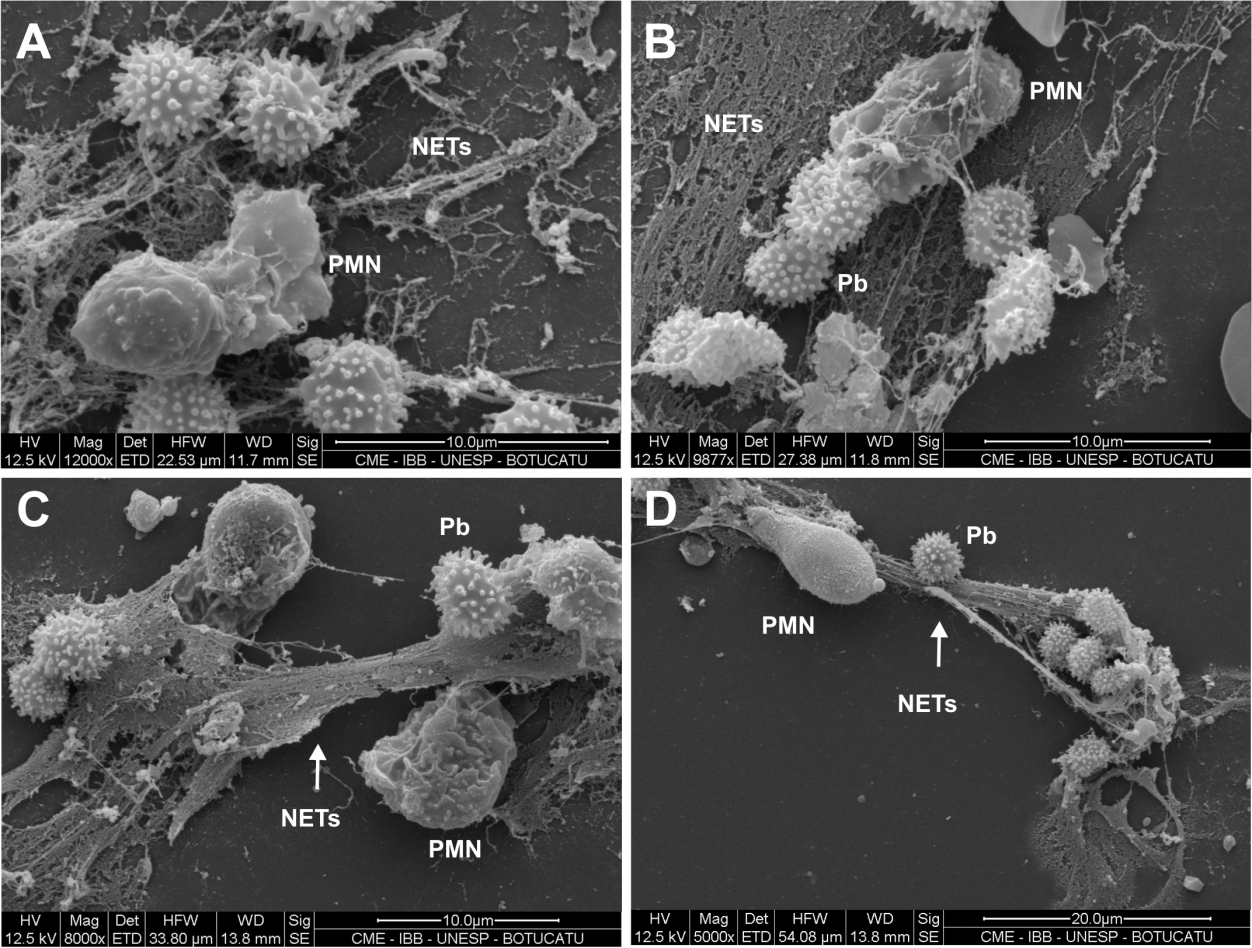
Scanning electron microscopy from patients’ PMNs challenged with Pb18 (50:1 ratio) (A and B) or Pb265 (50:1 ratio) (C and D). PMN cultures were challenged for two hours before analysis. (PMN–neutrophil; Pb–*P*. *brasiliensis*; NETs–Neutrophil Extracellular Traps).

Analyzing fungi-PMNs interaction, Pb18 was able to trigger NETs formation by PMNs in one and two hours of incubation (Fig 4B and 4C). Cultures with DNAse-1 treatment have not shown any evidence of NETs structures, confirming the enzyme’s degrading action upon the extracellular DNA structures (Fig 4D). We also observed that patient´s PMNs when challenged with both strains of *P*. *brasiliensis* (Pb18 and Pb265) released NETs in an attempt to entrap the fungi, possibly demonstrating the process of NETosis that may occur during *in vivo* infection with *P*. *brasiliensis* (Fig 5). However, distinct pattern of NETs in response to the different strains were detected. Analysis of cultures challenged with Pb18 revealed the presence of NETs in a large coverage area, and the structures seemed to be loose and scattered (Fig 5A and 5B). Whereas, Fig 5C and 5D demonstrated a different pattern of NETs when cells were challenged with Pb265. In these cultures, these structures looked smaller, denser and compacted when compared with Pb18 (Fig 5C and 5D). We observed that in Pb18 slides, practically all area was covered by NETs, while in Pb265 slides, NETs were concentrated near to the fungus.

### High-content image acquisition and quantification of NETs

**Fig 6:**
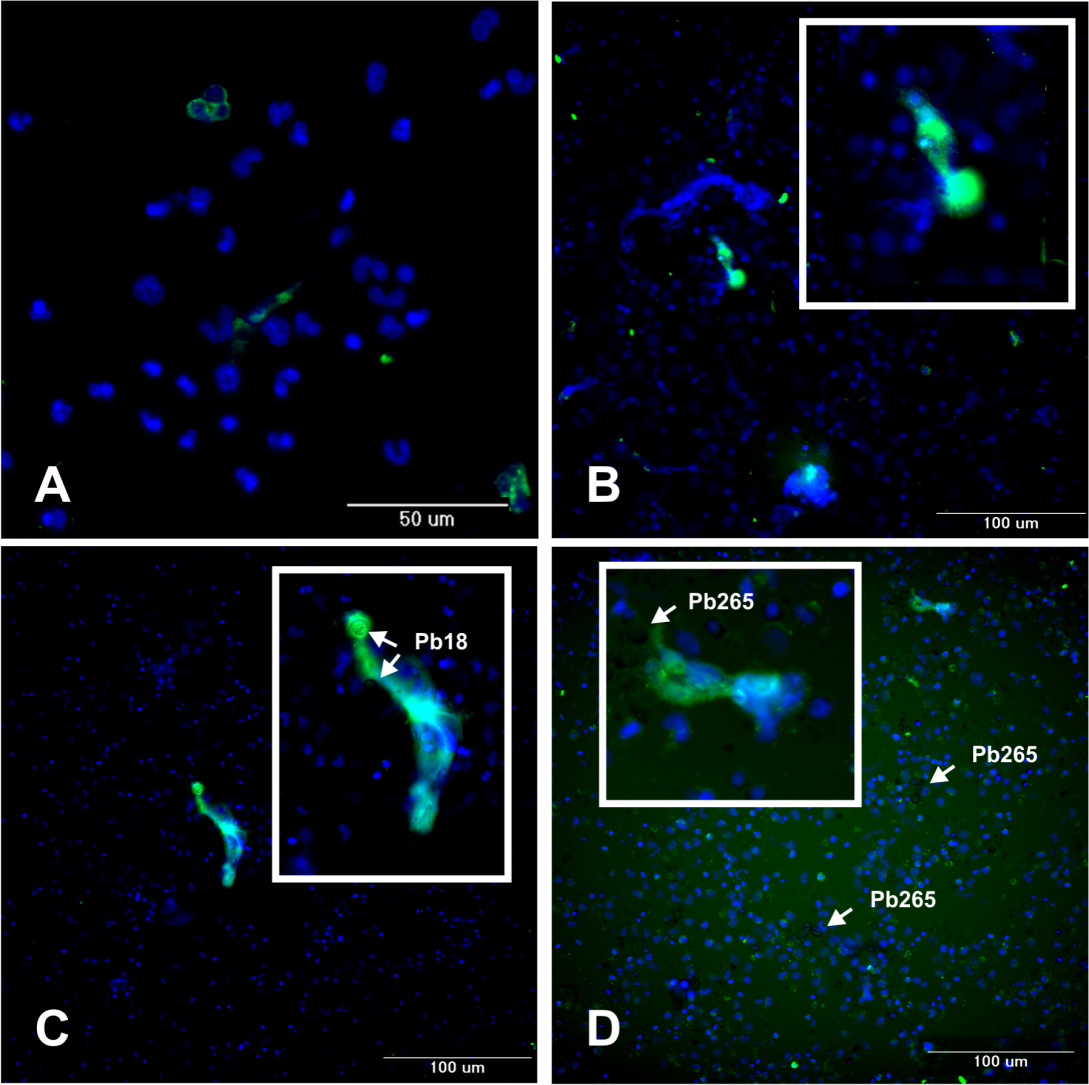
High-content image acquisition of PMN cultures from healthy donors stained with DAPI (DNA) and anti-elastase antibody (FITC-green). Non-treated PMNs (A), activated with PMA (100ng/mL) for 30 minutes (B), challenged with Pb18 (C) and with Pb265 (D) for two hours (50:1 ratio).

To corroborate data obtained by Confocal Microscopy and Scanning Electron Microscopy, we analyzed PMN cultures with the automated microscope ImageXpress Micro XL Widefield High-Content Screening System (Molecular Devices, Sunnyvale, CA). Once more, images showed an extracellular material stained with DAPI (DNA) and FITC (elastase), which characterized the presence of NETs in the cultures activated with PMA or challenged with Pb18 or Pb265 (Fig 6B, 6C and 6D, respectively). In Fig 6A, we observed non-treated PMNs, showing few PMNs in NETosis process, ratifying the idea that an activation is essential for NETs release. Whereas, in Fig 6B, when PMA was added to the culture, PMNs were able to release NETs to the extracellular environment. In Fig 6C and 6D, we observed Pb18 and Pb265, respectively, demonstrating the formation of NETs covering the yeast cells, certifying that this defense mechanism is triggered in the infection with *P*. *brasiliensis*.

**Fig 7:**
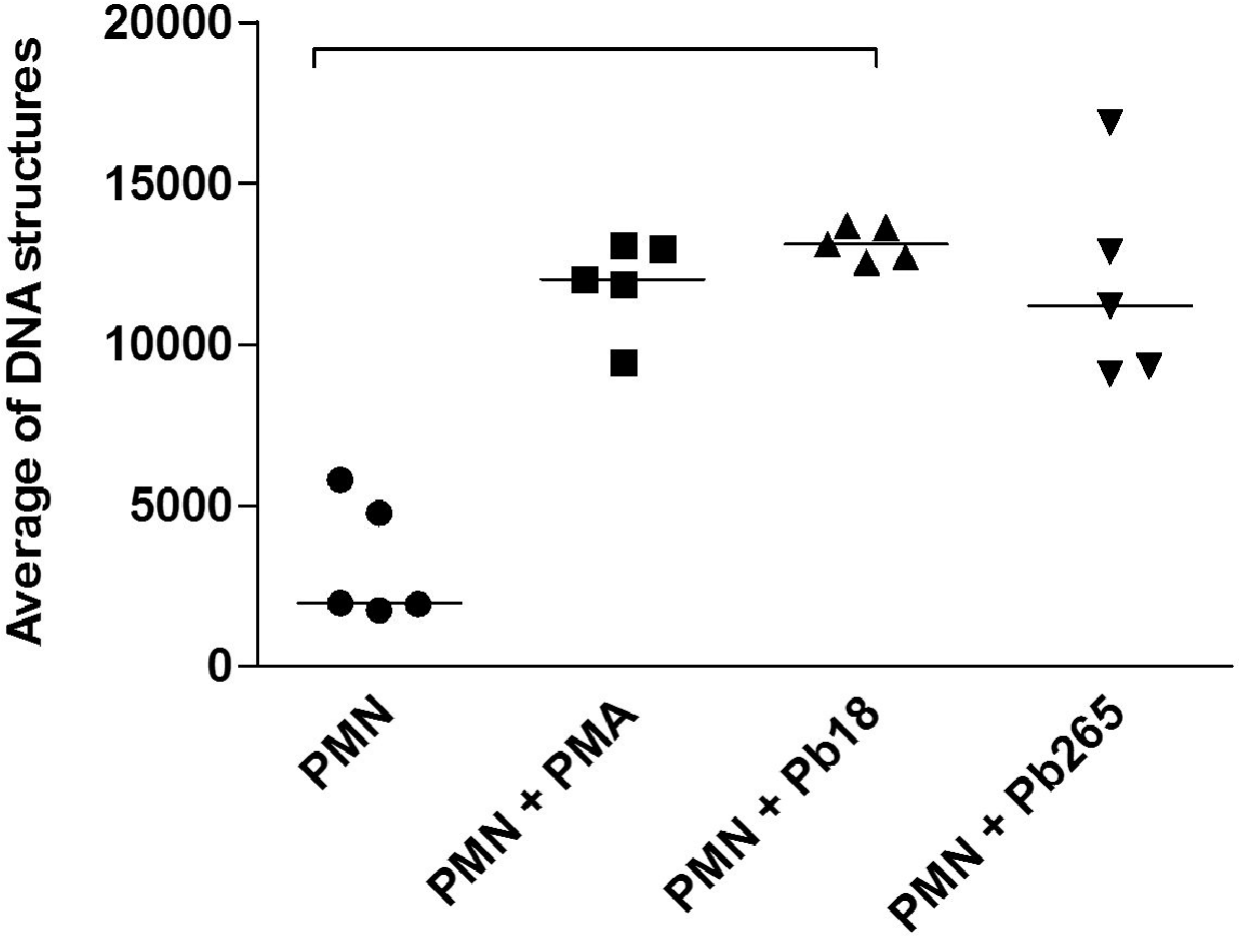
Average of total extracellular DNA structures released by PMN cultures activated with PMA (100 ng/mL) for 30 minutes or challenged with Pb18 or Pb265 (50:1 ratio) for two hours (p = 0.0016).

**Fig 8:**
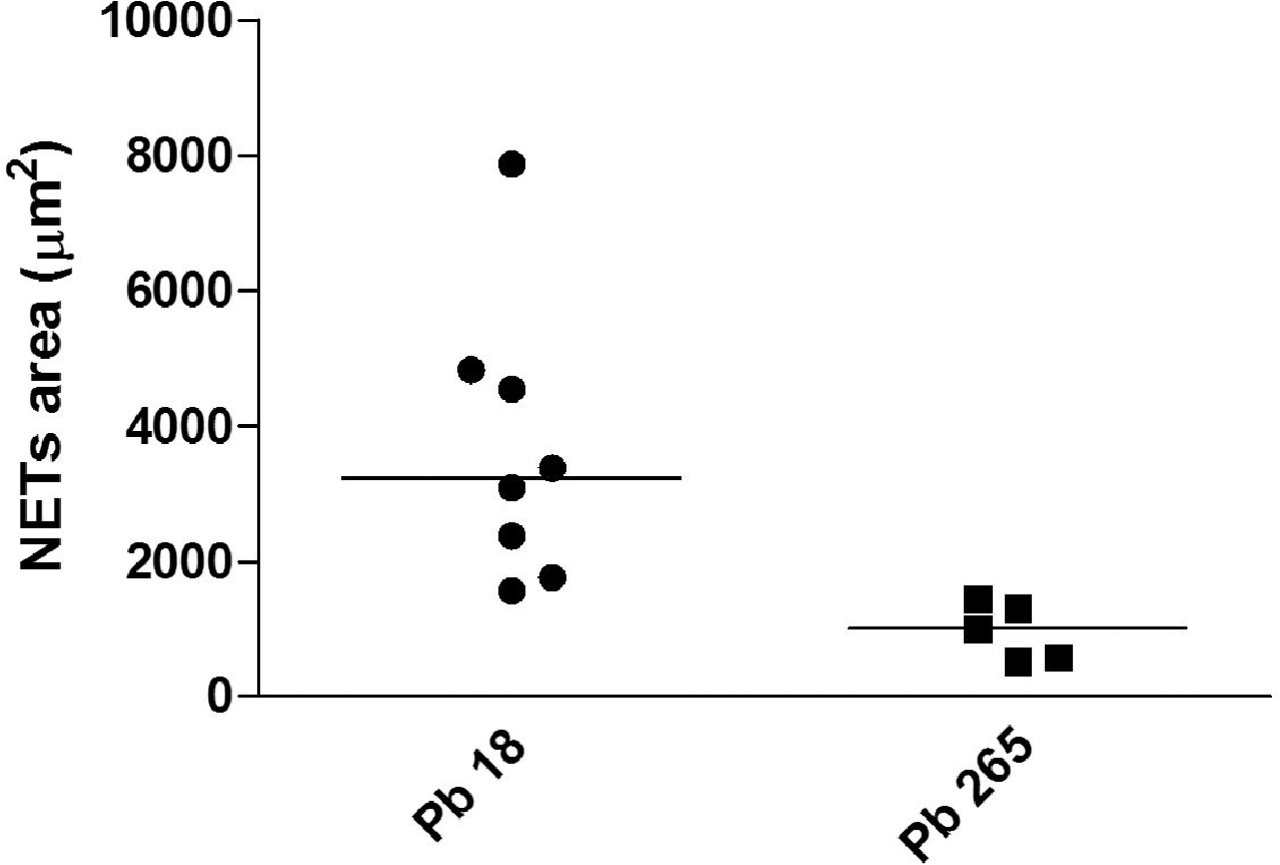
Measure of NETs area (μm^2^) released by PMN cultures challenged with Pb18 or Pb265 (50:1 ratio) for two hours (p = 0.0625).

**Fig 9:**
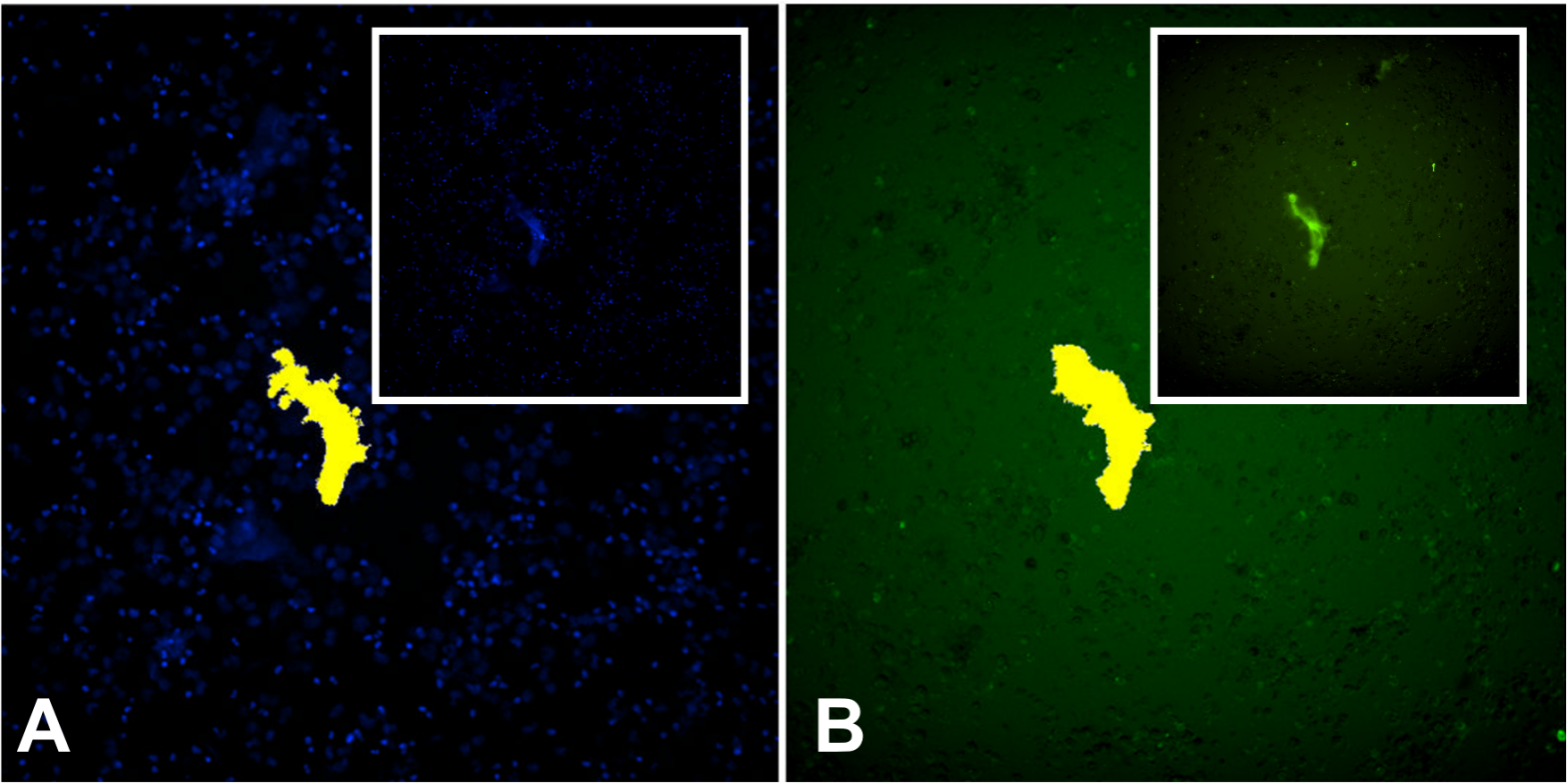
Image of the customized method with MetaXpress Custom Module Editor and measured by Integrated Morphometry Analysis. Images were acquired using the automated microscope ImageXpress Micro XL Widefield High-Content Screening System (Molecular Devices, Sunnyvale, CA) with a cooled 16-bit monochromes CMOS PCO camera (2160 × 2160 imaging array, 6.5 × 6.5 μm pixels) using an 20× Super Plan Fluor ELWD, NA 0.45 Nikon objective. Total area (μm^2^) of the resulting NETs in response to Pb18 and Pb265 was analyzed by thresholding the NETs stained with DAPI and anti-elastase-FITC antibody and measured by Integrated Morphometry Analysis. (A) DAPI and (B) FITC.

In addition to the obtained images, the same PMN cultures were analyzed in attempt to quantify the number of extracellular DNA structures, which represents NETs released after the culture’s challenge with Pb18, Pb265 or PMA activation. Analysis consisted in thresholding the structures stained with DAPI (DNA). Total DNA structures were quantified using a customized method with MetaXpress Custom Module Editor and measured by Integrated Morphometry Analysis presented as an average of extracellular DNA structures for each well. Corroborating our images, total extracellular DNA structures were substantially higher in the cultures activated with PMA or challenged with Pb18 or Pb265 when compared to non-treated PMNs, showing a significantly increase of extracellular DNA structures staining in cultures challenged mainly with Pb18 (p = 0.0016) (Fig 7). We also observed in these experiments, interesting different NETs patterns from cultures challenged with Pb18 or Pb265. Analyzing NETs area (μm^2^), that consisted in thresholding the structures stained with DAPI (DNA) colocalized with FITC (elastase), NETs from Pb18 coculture appeared to be bigger and more scattered than those presented by PMNs challenged with Pb265, which seemed to be smaller (p = 0.0625) (Fig 8), even though both are capable of entrapping the yeast cells as shown in Figs 5 and 6. Fig 9 was included to demonstrate how the software selected and performed the analysis.

**Fig 10:**
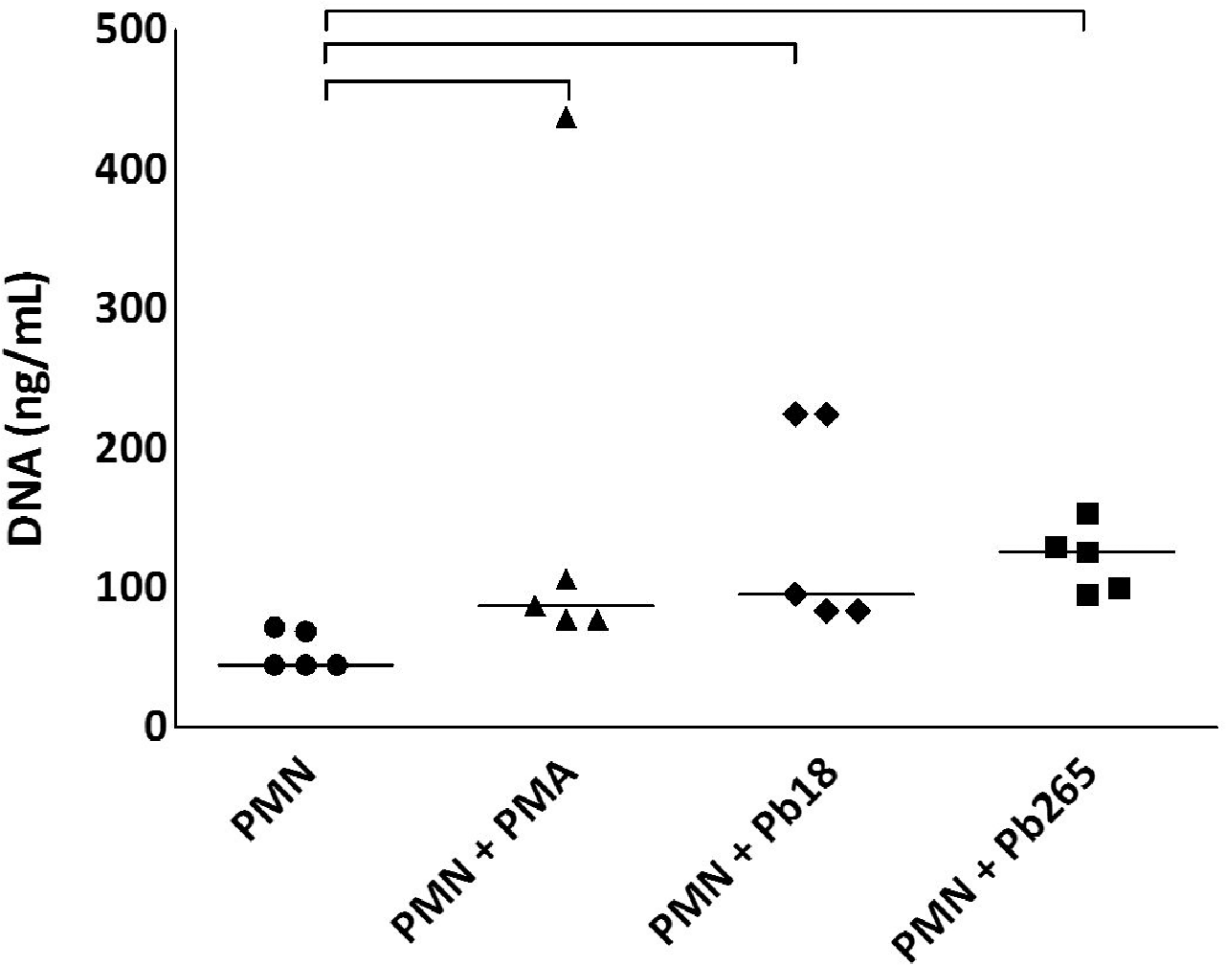
NETs quantification *in vitro*. PMNs from healthy donors were stimulated with PMA (100 ng/mL) for 30 minutes or challenged with Pb18 and Pb265 (50:1 ratio) for two hours. Supernatants were collected, and DNA were quantified by Picogreen dsDNA kit (p = 0.0167).

We also analyzed NETs through quantification of the extracellular DNA by Picogreen dsDNA kit (Invitrogen). PMNs from healthy donors were incubated for two hours with both strains of *P*. *brasiliensis* (Pb18 and Pb265). Corroborating the data shown in scanning electron microscopy and High-Content Screening System, both Pb18 and Pb265 were also able to induce release of NETs in the extracellular environment. However, there was no statistical difference between the two strains of *P*. *brasiliensis*. Non-treated PMNs released low levels of DNA when compared with cultures treated with PMA, Pb18 and Pb265 (p = 0.0167) (Fig 10).

## Discussion

NETs action has been widely studied over the recent years. Several microorganisms are able to induce formation and release of these structures and in some cases, as in infections caused by *Staphylococcus aureus*, *Shigella flexneri*, *Streptococcus pneumoniae*, *Leishmania amazonensis*, *Aspergillus fumigatus*, *Candida albicans*, *Aspergillus nidulans* and *P*. *brasiliensis*, these NETs have antimicrobial activities [28,31,34,37,38,43–47]. Recent study demonstrated that PMNs selectively responds to pathogens that are too large to be phagocytosed via NETosis process [48]. However, some pathogens have evasion mechanisms that make the entrapment only temporary, enabling the recruitment of other immune cells for the site or inhibiting the microorganisms’ growth, as seeing in other infections [33,35–37,45,49].

The interaction between NETs and *P*. *brasiliensis* is still been investigated. In this study, we demonstrated for the first time NETs formation *in vivo* induced by *P*. *brasiliensis* yeast cells in tegumentary lesions of patients. Our results *in vitro* showed that yeasts are trapped by NETs, corroborating our previous study, in which were evidenced that *P*. *brasiliensis* yeast cells are able to induce NETs formation by PMNs and that these structures are involved in extracellular killing of the fungus [43]. However, our results also demonstrated different patterns of NETs in a dependence of the evaluated strain.

This study identified NETs components, such as histone and elastase, in both analyzes, in patient's tegumentary lesions and in PMN cultures, by confocal laser scanning microscopy and High-Content Screening System. Furthermore, it was identified in some tissue sections, cells in the initial process of NETs formation, demonstrating the colocalization of nuclear DNA with elastase, which is transported to the nucleus and acts on histones to initiate the chromatin decondensation, even before it is released into the cytoplasm. Papayannopoulos *et al*. [42] showed that elastase is necessary for chromatin decondensation through degradation of histones, allowing NETs release to the extracellular environment. Thus, we believe that the weak labeling of histone on this image was due the elastase action upon this component (Fig 3). Interestingly, the decondensed nuclear material labeled with DAPI that appears to be released by cells in some images, still have certain conserved nuclear structure. This is consistent with what has been recently proposed by some authors, that NETs can be formed by viable PMNs and that cell death can occur subsequently, once cell death is not a requirement for the formation of such structures [50–52].

Histopathological analysis was performed using H&E and Gomori-Grocott stainings and was included in this study in an attempt to identify structures that could indicate the presence of yeast cells in the local of NETs formation, characterized by basophilic material with extracellular filaments, indicating extracellular DNA positive for hematoxylin. Urban *et al*. [38] also identified the presence of NETs in histopathological analysis of mice challenged with *C*. *albicans*, evidencing the presence of extracellular DNA positive for hematoxylin, as seen in this study, corroborating once more our findings on the lesion analysis by confocal laser scanning microscopy. In this manner, the presence of NETs in tegumentary lesions strengthens the results obtained in the *in vitro* experiments, proving that there is a release of these NETs against the fungus after PMNs recruitment *in vivo*, once *P*. *brasiliensis* was identified in the lesion.

Scanning electron microscopy images showed similar structures as those first identified by Brinkmann *et al*. [28] and others [31,33,46]. In our images, the interaction between PMNs and *P*. *brasiliensis* yeast cells was evidenced, with consequent release of net like material, suggesting a role of NETs during PCM. These structures were similar to those seen in other microbe-NETs interaction like *Mycobacterium tuberculosis*, *L*. *amazonensis and P*. *brasiliensis* [33,34,43] and to images presented in previous studies with other fungi such as *C*. *albicans*, *A*. *fumigatus*, *A*. *nidulans* and *Cryptococcus gattii* [31,37,38,46,47,53].

However, when cells were challenged with different strains of *P*. *brasiliensis*, we identified a distinct pattern of NETs in a dependence of the evaluated strain. PMN cultures challenged with Pb18 showed presence of NETs in a large coverage area and these structures seemed to be loose and scattered, whereas NETs observed in PMN cultures challenged with Pb265 appeared to be smaller, denser and compacted, as shown in Scanning Electron Microscopy and by High Content analysis. We believe that this different NETs pattern might be related to an enzyme with DNAse like activity produced by Pb18 strain as a possible escaping mechanism avoiding a tight entrapment by NETs, which could contribute to the observed pattern. As demonstrated, in Pb18 genome (ABKI00000000.2) [54], there are two hypothetical proteins (PADG_11161—Gene ID: 22587058 and PADG_08285—Gene ID: 22586608) that have DNAse activities. Besides, it was already identified a deoxyribonuclease gene (PAAG_07587—Gene ID: 9093585) in P. lutzzi genome (ABKH00000000.2) [54]. Studies to better explain these hypothesis are been conducted in our lab. Beyond that, Buchanan (2006) have shown the production of DNAse by group A *Streptococcus* as an escaping mechanism from NETosis [55].

Although NETs have two important effector functions against microorganisms, as temporary imprison of the pathogen that prevents its spread and a direct antimicrobial action on trapped microorganisms, studies have also related NETs with several diseases, correlating them with pathological effects. Some reports show that antimicrobial histones and peptides coating the NET-DNA have direct cytotoxic effect to tissue, and ineffective clearance of NETs is responsible for deleterious inflammation of host tissue in several disorders [41,56–64]. In acute respiratory distress syndrome (ARDS), there is a massive influx of PMNs into the lungs causing neutrophilic inflammation and in acute lung injury (ALI), there is an excessive activation and migration of PMNs into the lung. These cells are important contributors to the progression of ALI/ARDS, and higher PMN concentration in the bronchoalveolar lavage (BAL) fluid of patients with ARDS is often associated with greater severity of the disease [64,65], while excessive PMNs and NETs contribute to the pathology of ALI, where NETs can directly induce lung epithelial cell death [62], relating NETs to the pathogenesis of these important lung diseases [57,59,60], as described above.

Therefore, this mechanism could also be involved with the pathogenesis of PCM, once it was described the presence of PMNs in inflammatory infiltrates of granulomas and in lesions detected in disease experimental models [66–70] and now the evidence of NETs in the lesions. Thus, the real participation of NETs in defense against *P*. *brasiliensis* or in disease’s pathogenesis, as well as the fungal escape mechanisms involved needs to be further investigated.

In conclusion, our data show for the first time the identification of NETs in patients’ tegumentary lesions. Beyond that, both strains of *P*. *brasiliensis* (Pb18 and Pb265) are able to induce NETs formation by human PMNs *in vitro* and are related to different patterns of NETs. The presence of NETs components both *in vivo* and *in vitro* open new possibilities for the detailed investigation of the immunity in PCM.

## References

[1] Ginarte M, Pereiro M Jr, Toribio J. Imported paracoccidioidomycosis in Spain. Mycoses. 2003; 46(9–10):407–411. 14622390 10.1046/j.0933-7407.2003.00914.x

[2] Van Damme PA, Bierenbroodspot F, Telgtt DS, Kwakman JM, De Wilde PC, Meis JF. A case of imported paracoccidioidomycosis: an awkward infection in The Netherlands. Med Mycol. 2006 2; 44(1):13–18. 16805088 10.1080/13693780500148137

[3] Bousquet A, Dussart C, Drouillard I, Charbel EC, Boiron P. Imported mycosis: a review of paracoccidioidomycosis. Med Mal Infect. 2007 12; 37 Suppl 3:S210–214. 17988812 10.1016/j.medmal.2007.09.007

[4] Mayayo E, López-Aracil V, Fernández-Torres B, Mayayo R, Domínguez M. Report of an imported cutaneous disseminated case of paracoccidioidomycosis. Rev Iberoam Micol. 2007 3; 24(1):44–46. 17592892 10.1016/s1130-1406(07)70011-6

[5] Alsharif M, Martin AU, Shelton JB Jr, Pambuccian SE. *Paracoccidioides brasiliensis* in a liquid-based Papanicolaou test from a pregnant woman: report of a case. Diagn Cytopathol. 2008 8; 36(8):557–560. 10.1002/dc.20847 18618723

[6] Matute DR, McEwen JG, Puccia R, Montes BA, San-Blas G, Bagagli E, et al Cryptic speciation and recombination in the fungus *Paracoccidioides brasiliensis* as revealed by gene genealogies. Mol Biol Evol. 2006 1; 23(1):65–73. 16151188 10.1093/molbev/msj008

[7] Teixeira MM, Theodoro RC, Carvalho MJ, Fernandes L, Paes HC, Hahn RC, et al Phylogenetic analysis reveals a high level of speciation in the *Paracoccidioides* genus. Mol Phylogenet Evol. 2009 8; 52(2):273–283. 10.1016/j.ympev.2009.04.005 19376249

[8] Restrepo A and Tobón AM. Paracoccidioides brasiliensis In: Principles and Practice of Infectious Diseases. Elsevier 2005; 3062–3068.

[9] Marques SA. Paracoccidioidomycosis: epidemiological, clinical, diagnostic and treatment up-dating. An Bras Dermatol. 2013 Sep-Oct; 88(5):700–711. 10.1590/abd1806-4841.20132463 24173174 PMC3798345

[10] da Silva Neto BR, Carvalho PFZ, Bailão AM, Martins WS, Soares CMA, Pereira M. Transcriptional profile of *Paracoccidioides* spp. In response to itraconazole. BMC Genomics. 2014 4 1; 15:254 10.1186/1471-2164-15-254 24690401 PMC3975141

[11] Franco M, Peraçoli MTS, Soares AMVC, Montenegro R, Mendes RP, Meira DA. Host-parasite relationship in paracoccidioidomycosis. Curr Top Med Mycol. 1993; 5:115–149. 8242798

[12] Nicola AM, Casadevall A, Goldman DL. Fungal killing by mammalian phagocytic cells. Curr Opin Microbiol. 2008 8; 11(4):313–317. 10.1016/j.mib.2008.05.011 18573683 PMC2563425

[13] do Nascimento MP, de Campos Soares AM, Dias-Melicio LA, Parise-Fortes MR, Martins RA, Nakaira ET, et al Fungicidal activity of human monocyte-derived multinucleated giant cells induced in vitro by *Paracoccidioides brasiliensis* antigen. Mycopathologia. 2008 7; 166(1):25–33. 10.1007/s11046-007-9051-6 18496765

[14] Moreira AP, Dias-Melicio LA, Soares AMVC . Interleukin-10 but not Transforming Growth Factor beta inhibits murine activated macrophages *Paracoccidioides brasiliensis* killing: effect on H2O2 and NO production. Cell Immunol. 2010; 263(2):196–203. 10.1016/j.cellimm.2010.03.016 20417928

[15] Bordon-Graciani Ap, Dias-Melicio LA, Acorci-Valerio MJ, Araujo JP Jr, de Campos Soares AM. Inhibitory effect of PGE 2 on the killing of *Paracoccidioides brasiliensis* by human monocytes can be reversed by cellular activation with cytokines. Med Mycol. 2012 10; 50(7):726–734. 10.3109/13693786.2012.676740 22548241

[16] Rodrigues DR, Fernandes RK, Balderramas HA, Penitenti M, Bachiega TF, Calvi SA, et al Interferon-gamma production by human neutrophils upon stimulation by IL-12, IL-15 and IL-18 and challenge with *Paracoccidioides brasiliensis* . Cytokine. 2014 9; 69(1):102–109. 10.1016/j.cyto.2014.05.009 25022968

[17] Balderramas HA, Penitenti M, Rodrigues DR, Bachiega TF, Fernandes RK, Ikoma MR, et al Human neutrophils produce IL-12, IL-10, PGE2 and LTB4 in response to *Paracoccidioides brasiliensis*. Involvment of TLR2, mannose receptor and dectin-1. Cytokine. 2014 5; 67(1):36–43. 10.1016/j.cyto.2014.02.004 24680480

[18] Souto JT, Aliberti JC, Campanelli AP, Livonesi MC, Maffei CM, Ferreira BR, et al Chemokine production and leukocyte recruitment to the lungs of *Paracoccidioides brasiliensis* infect mice is modulated by interferon-gamma. Am J Pathol. 2003 8; 163(2):583–590. 12875978 10.1016/s0002-9440(10)63686-3 PMC1868217

[19] Segal AW. How Neutrophils Kill Microbes. Annu Rev Immunol. 2005; 23:197–223. 15771570 10.1146/annurev.immunol.23.021704.115653 PMC2092448

[20] Moreira AP, Dias-Melicio LA, Peraçoli MT, Calvi SA, Victoriano de Campos Soares AM. Killing of *Paracoccidioides brasiliensis* yeast cells by IFN-gamma and TNF-alpha activated murine peritoneal macrophages: evidence of H(2)O(2) and NO effector mechanisms. Mycopathologia. 2008 7; 166(1):17–23. 10.1007/s11046-007-9046-3 18496766

[21] Carmo JP, Dias-Melicio LA, Calvi SA, Peraçoli MT, Soares AM . TNF-alpha activates human monocytes for *Paracoccidioides brasiliensis* killing by an H2O2-dependent mechanism. Med Mycol. 2006 6; 44(4):363–368. 16772231 10.1080/13693780500536885

[22] Costa DL, Dias-Melicio LA, Acorci MJ, Bordon AP, Tavian EG, Peraçoli MT, et al Effect of interleukin-10 on the *Paracoccidioides brasiliensis* by gamma-interferon activated human neutrophils. Microbiol Immunol. 2007; 51(1):73–80. 17237601 10.1111/j.1348-0421.2007.tb03892.x

[23] Bannwart CF, Martins RA, Nakaira-Takahashi E, Dias-Melicio LA, Soares AM, Peraçoli MT. Interleukin-15 augments oxidative metabolism and fungicidal activity of human monocytes against *Paracoccidioides brasiliensis* . Mem Inst Oswaldo Cruz. 2010 11; 105(7):866–872. 21120355 10.1590/s0074-02762010000700005

[24] Kurita N, Oarada M, Ito E, Miyaji M. Antifungal activity of human polymorphonuclear leucocytes against yeast cells of *Paracoccidioides brasiliensis* . Med Mycol. 1999 8; 37(4):261–267. 10421861

[25] Kurita N, Oarada M, Miyaji M, Ito E. Effect of cytokines on antifungal activity of human polymorphonuclear leucocytes against yeast cells of *Paracoccidioides brasiliensis* . Med Mycol. 2000 4; 38(2):177–182. 10817235 10.1080/mmy.38.2.177.182

[26] Rodrigues DR, Dias-Melicio LA, Calvi AS, Peraçoli MT, Soares AM. *Paracoccidioides brasiliensis* killing by IFN-gamma, TNF-alpha and GM-CSF activated human neutrophils: role for oxygen metabolites. Med Mycol. 2007 2; 45(1):27–33. 17325941 10.1080/13693780600981676

[27] Tavian EG, Dias-Melicio LA, Acorci MJ, Graciani AP, Peraçoli MT, Soares AM. Interleukin-15 increases *Paracoccidioides brasiliensis* killing by human neutrophils. Cytokine. 2008 1; 41(1):48–53. 18086532 10.1016/j.cyto.2007.10.011

[28] Brinkmann V, Reichard U, Goosmann C, Fauler B, Uhlemann Y, Weiss DS, et al Neutrophil extracellular traps kill bacteria. Science. 2004 3 5; 303(5663):1532–1535. 15001782 10.1126/science.1092385

[29] von Köckritz-Blickwede M, Nizet V. Innate immunity turned inside-out: antimicrobial defense by phagocyte extracellular traps. J Mol Med (Berl). 2009 8; 87(8):775–783. 19444424 10.1007/s00109-009-0481-0 PMC2707954

[30] Martinelli S, Urosevic M, Daryadel A, Oberholzer PA, Baumann C, Fey MF, et al Induction of genes mediating interferon-dependent extracellular trap formation during neutrophil differenciation. J Biol Chem. 2004 10 15; 279(42):44123–44132. 15302890 10.1074/jbc.M405883200

[31] Urban CF, Reichard U, Brinkmann V, Zychlinsky A. Neutrophil extracellular traps capture and kill *Candida albicans* yeast and hyphal forms. Cell Microbiol. 2006 4; 8(4):668–676. 16548892 10.1111/j.1462-5822.2005.00659.x

[32] Grinberg N, Elazar S, Rosenshine I, Shpigel NY. Beta-hydroxybutyrate abrogates formation of bovine neutrophil extracellular traps and bactericidal activity against mammary pathogenic *Escherichia coli* . Infect Immun. 2008 6; 76(6):2802–2807. 10.1128/IAI.00051-08 18411287 PMC2423099

[33] Ramos-Kichik V, Mondragon-Flores R, Mondragon-Castelan M, Gonzalez-Pozos S, Muñiz-Hernandez S, Rojas-Espinosa O, et al Neutrophil extracellular traps are induced by *Mycobacterium tuberculosis* . Tuberculosis (Edinb). 2009 1; 89(1):29–37. 19056316 10.1016/j.tube.2008.09.009

[34] Guimarães-Costa AB, Nascimento MTC, Froment GS, Soares RP, Morgado FN, Conceição-Silva S, et al *Leishmania amazonensis* promastigotes induce and are killed by neutrophil extracellular traps. Proc Natl Acad Sci USA. 2009 4 21; 106(16):6748–6753. 10.1073/pnas.0900226106 19346483 PMC2672475

[35] Menegazzi R, Decleva E, Dri P. Killing by neutrophil extracellular traps: fact or folklore? Blood. 2012 2 2; 119(5):1214–1216. 10.1182/blood-2011-07-364604 22210873

[36] Malachowa N, Kobayashi SD, Freedman B, Doward DW, DeLeo FR. *Staphylococcus aureus* Leukotoxin GH promotes formation of Neutrophil Extracellular Traps. J Immunol. 2013 12 15; 191(12):6022–6029. 10.4049/jimmunol.1301821 24190656 PMC3903389

[37] McCormick A, Heesemann L, Wagener J, Marcos V, Hartl D, Loeffler J, et al NETs formed by human neutrophils inhibit growth of the pathogenic mold *Aspergillus fumigatus* . Microbes Infect. 2010 11; 12(12–13):928–936. 10.1016/j.micinf.2010.06.009 20603224

[38] Urban CF, Ermert D, Schmid M, Abu-Abed U, Goosmann C, Nacken W, et al Neutrophil extracellular traps contain calprotectin, a cytosolic protein complex involved in host defense against *Candida albicans* . PLoS Pathog. 2009 10; 5(10): e1000639 10.1371/journal.ppat.1000639 19876394 PMC2763347

[39] Gabriel C, McMaster WR, Girard D, Descoteaux A. *Leishmania donovani* promastigotes evade the antimicrobial activity of neutrophil extracellular traps. J Immunol. 2010 10 1; 185(7):4319–4327. 10.4049/jimmunol.1000893 20826753

[40] Byrd AS, O´Brien XM, Johnson CM, Lavigne LM, Reichner JS. An extracellular matrix based mechanism of rapid neutrophil extracellular trap formation in response to *Candida albicans* . J Immunol. 2013 4 15; 190(8):4136–4148. 10.4049/jimmunol.1202671 23509360 PMC3622194

[41] Leffler J, Martin M, Gullstrand B, Tydén H, Lood C, Truedsson L, et al Neutrophil extracellular traps that are not degraded in systemic lupus erythematosus activate complement exacerbating the disease. J Immunol. 2012 4 1; 188(7):3522–3531. 10.4049/jimmunol.1102404 22345666

[42] Papayannopoulos V, Metzler KD, Hakkim A, Zychlinsky A. Neutrophil elastase and myeloperoxidase regulate the formation of neutrophil extracellular traps. J Cell Biol. 2010 11 1; 191(3):677–691. 10.1083/jcb.201006052 20974816 PMC3003309

[43] Bachiega TF, Fernandes RK, Rodrigues DR, Balderramas HA, Dias-Melicio LA, Soares AMVC . *Paracoccidioides brasiliensis* induces neutrophil extracellular traps in vitro. Front Immunol 2013.

[44] Mejía SP, Cano LE, López JA, Hernandez O, González Á. Human neutrophils produce extracellular traps against *Paracoccidioides brasiliensis* . Microbiology. 2015 5; 161(Pt 5):1008–1017. 10.1099/mic.0.000059 25701733

[45] Wartha F, Beiter K Albiger B, Fernebro J, Zychlinsky A, Normark S, et al Capsule and D-alanylated lipoteichoic acids protects *Streptococcus pneumoniae* against neutrophil extracellular traps. Cell Microbiol. 2007 5; 9(5):1162–1171. 17217430 10.1111/j.1462-5822.2006.00857.x

[46] Bruns S, Kniemeyer O, Hasenberg M, Aimanianda V, Nietzsche S, Thywissen A, et al Production of extracellular traps against *Aspergillus fumigatus* in vitro and in infected lung tissue is dependent on invading neutrophils and influenced by hydrophobin RodA. PLoS Pathog. 2010 4 29; 6(4):e1000873 10.1371/journal.ppat.1000873 20442864 PMC2861696

[47] Bianchi M, Hakkim A, Brinkmann V, Siler U, Seger RA, Zychlinsky A, et al Restoration of NET formation by gene therapy in CGD controls aspergillosis. Blood. 2009 9 24; 114(13):2619–2622. 10.1182/blood-2009-05-221606 19541821 PMC2756123

[48] Branzk N, Lubojemska A, Hardison SE, Wang Q, Gutierrez MG, Brown GD, et al Neutrophils sense microbe size and selectively release neutrophil extracellular traps in response to large pathogens. Nat Immunol. 2014 11; 15(11):1017–1025. 10.1038/ni.2987 25217981 PMC4236687

[49] Guimarães-Costa AB, De Souza-Vieira TS, Paletta-Silva R, Freitas-Mesquita AL, Meyer-Fernandes JR, Saraiva EM . 3'-nucleotidase/nuclease activity allows *Leishmania* parasites to escape killing by neutrophil extracellular traps. Infect Immun. 2014 4; 82(4):1732–1740. 10.1128/IAI.01232-13 24516114 PMC3993383

[50] Clark SR, Ma AC, Tavener SA, McDonald B, Goodarzi Z, Kelly MM, et al Platelet TLR4 activates neutrophil extracellular traps to ensnare bacteria in septic blood. Nat Med. 2007 4; 13(4):463–469. 17384648 10.1038/nm1565

[51] Pilsczek FH, Salina D, Poon KK, Fahey C, Yipp BG, Sibley CD, et al A novel mechanism of rapid nuclear neutrophil extracellular trap formation in response to *Staphylococcus aureus* . J Immunol. 2010 12 15; 185(12):7413–7425. 10.4049/jimmunol.1000675 21098229

[52] Simon D, Simon HU, Yousefi S. Extracellular DNA traps in allergic, infectious, and autoimmune diseases. Allergy. 2013 4; 68(4):409–416. 10.1111/all.12111 23409745

[53] Springer DJ, Chaturvedi V. Projecting global occurrence of *Cryptococcus gattii* . Emerg Infect Dis. 2010 1; 16(1):14–20. 10.3201/eid1601.090369 20031037 PMC2874352

[54] Desjardins CA, Champion MD, Holder JW, Muszewska A, Goldberg J, Bailão AM, et al Comparative genomic analysis of human fungal pathogens causing paracoccidioidomycosis. PLoS Genet. 2011 10; 7(10):e1002345 10.1371/journal.pgen.1002345 22046142 PMC3203195

[55] Buchanan JT, Simpson AJ, Aziz RK, Liu GY, Kristian SA, Kotb M, et al DNAse expression allows the pathogen group A *Streptococcus* to escape killing in neutrophil extracellular traps. Curr Biol. 2006 2 21; 16(4): 396–400. 16488874 10.1016/j.cub.2005.12.039

[56] Xu J, Zhang X, Pelayo R, Monestier M, Ammollo CT, Semararo F, et al Extracellular histones are major mediators of cell death in sepsis. Nat Med. 2009 11; 15(11):1318–1321. 10.1038/nm.2053 19855397 PMC2783754

[57] Döring Y, Manthey HD, Drechsler M, Lievens D, Megens RT, Soehnlein O, et al Auto-antigenic protein-DNA complexes stimulate plasmocytoid dendritic cells to promote atherosclerosis. Circulation. 2012 4 3; 125(13):1673–1683. 10.1161/CIRCULATIONAHA.111.046755 22388324

[58] Kessenbrock K, Krumbholz M, Schönermarck U, Back W, Gross WL, Werb Z, et al Netting neutrophils in autoimmune small-vessel vasculitis. Nat Med. 2009 6; 15(6):623–625. 10.1038/nm.1959 19448636 PMC2760083

[59] Hakkim A, Fürnrohr BG, Amann K, Laube B, Abed UA, Brinkmann V, et al Impairment of neutrophil extracellular trap degradation is associated with lupus nephritis. Proc Natl Acad Sci USA. 2010 5 25; 107(21):9813–9818. 10.1073/pnas.0909927107 20439745 PMC2906830

[60] Lande R, Ganguly D, Facchinetti V, Frasca L, Conrad C, Gregorio J, et al Neutrophils activate plasmocytoid dendritic cells by releasing self-DNA-peptide complexes in systemic lupus erythematosus. Sci Transl Med. 2011 3 9; 3(73):73ra19 10.1126/scitranslmed.3001180 21389263 PMC3399524

[61] Liu CL, Tangsombatvisit S, Rosenberg JM, Mandelbaum G, Gillespie EC, Gozani OP, et al Specific post-translational histone modifications of neutrophil extracellular traps as immunogens and potential targets of lupus autoantibodies. Arthritis Res Ther. 2012 2 2; 14(1):R25 10.1186/ar3707 22300536 PMC3392818

[62] Saffarzadeh M, Juenemann C, Queisser MA, Lochnit G, Barreto G, Galuska SP, et al Neutrophil extracellular traps directly induce epithelial and endothelial cell death: a predominant role of histones. PLoS One. 2012; 7(2):e32366 10.1371/journal.pone.0032366 22389696 PMC3289648

[63] Rohrbach AS, Hemmers S, Arandjelovic S, Corr M, Mowen KA. PAD4 is not essential for disease in the K/BxN murine autoantibody-mediated model of arthritis. Arthritis Res Ther. 2012 5 2; 14(3):R104 10.1186/ar3829 22551352 PMC3446481

[64] Cheng OZ, Palaniyar N . NET balancing: a problem in inflammatory lung diseases . Front Immunol. 2013 1 24; 4:1. 10.3389/fimmu.2013.00001 PMC3553399 23355837

[65] Grommes J, Soehnlein O. Contributions of neutrophils to acute lung injury. Mol Med. 2011 Mar-Apr; 17(3–4):293–307. 10.2119/molmed.2010.00138 21046059 PMC3060975

[66] Uribe F, Zuluaga AI, León W, Restrepo A. Histopathology of cutaneous and mucosal lesions in human paracoccidioidomycosis. Rev Inst Med Trop Sao Paulo. 1987 Mar-Apr; 29(2):90–96. 3423617 10.1590/s0036-46651987000200005

[67] Araújo VC, Demasi AP, Soares AB, Passador-Santos F, Napimoga MH, Martinez EF, et al Neutrophils in oral paracoccidioidomycosis and the involvement of Nrf2. PLoS One. 2013 10 24; 8(10): e76976 10.1371/journal.pone.0076976 24204715 PMC3811996

[68] De Faveri J, Coelho KI, Rezkallah-Iwasso MT, Franco M . Hypersensitivity pneumonitis to Paracoccidioides brasiliensis antigens in mice. J Med Vet Mycol. 1989; 27(2): 93–104 2501469

[69] De Faveri J, Rezkallah-Iwasso MT, Franco MF. Pulmonary paracoccidioidomycosis in immunized mice. Mycopathologia. 1992 7; 119(1):1–9. 1406901 10.1007/BF00492223

[70] Lopera D, Naranjo TW, Cruz OG, Restrepo A, Cano LE, Lenzi HL. Structural and topographic dynamics of pulmonary histopathology and local cytokine profiles in Paracoccidioides brasiliensis conidia-infected mice. PLoS Negl Trop Dis. 2011 7;5(7):e1232 10.1371/journal.pntd.0001232 21765962 PMC3134433

